# Supplementary material for: Anti-Inflammatory Activity of Fucoidan Extracts In Vitro
Source: Mar Drugs. 2021 Dec 11;19(12):702. doi: 10.3390/md19120702 (PMC8704339; doi:10.3390/md19120702)
Supplement: Supplementary file 1 [file marinedrugs-19-00702-s001.zip › marinedrugs-1441271-supplementary.pdf]

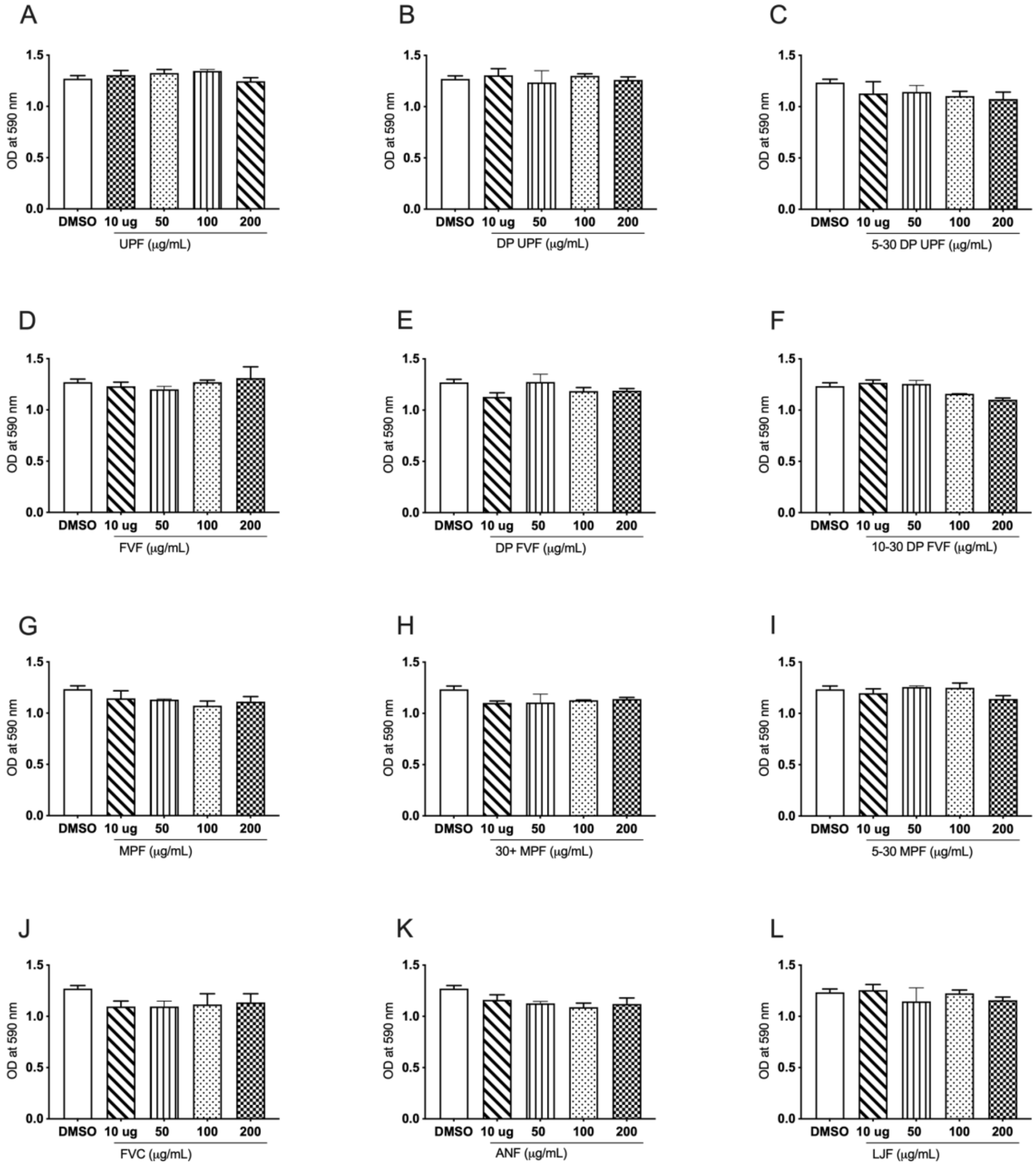

Suppementary Figure S1

Effects of fucoidan extracts on THP-1 cell viability. Cells were treated with the specified concentrations of fucoidan extract for 48 h and assessed by MTT reduction assays. Results expressed OD of treated cells vs vehicle control. Data represented as means ± SEM. Statistical analysis were carried out using one-way ANOVA followed by Tukey's multiple comparison test. A p-value of <0.05 is considered significant if applicable.
